# A Bayesian Perspective on Sensory and Cognitive Integration in Pain Perception and Placebo Analgesia

**DOI:** 10.1371/journal.pone.0117270

**Published:** 2015-02-09

**Authors:** Davide Anchisi, Marco Zanon

**Affiliations:** Department of Medical and Biological Sciences, Universit degli Studi di Udine, Udine, Italy; Brain and Spine Institute (ICM), FRANCE

## Abstract

The placebo effect is a component of any response to a treatment (effective or inert), but we still ignore why it exists. We propose that placebo analgesia is a facet of pain perception, others being the modulating effects of emotions, cognition and past experience, and we suggest that a computational understanding of pain may provide a unifying explanation of these phenomena. Here we show how Bayesian decision theory can account for such features and we describe a model of pain that we tested against experimental data. Our model not only agrees with placebo analgesia, but also predicts that learning can affect pain perception in other unexpected ways, which experimental evidence supports. Finally, the model can also reflect the strategies used by pain perception, showing that modulation by disparate factors is intrinsic to the pain process.

## Introduction

How can an inert treatment cause a response? Although a growing number of studies have addressed the placebo effect, which is the response following an inert treatment administered as if it were real, an explanation of its basic functioning is far from complete. Various hypotheses attribute the effect to the expectation of the subject and/or to implicit conditioning toward the context associated with the treatment, while experimental evidences show that this effect also contributes to the response to active treatments [1–4]. Recent research has identified some of the neurobiology underlying the placebo effect [1, 5–8], such as the role of antinociceptive descending pathways and endogenous opioids in placebo analgesia [9, 10]. Despite these advancements, some fundamental questions remain [2]. For example we still do not know why the placebo effect exists, or why a person has to trust that a treatment is going to work to recruit this endogenous antinociceptive capability.

Here we suggest that a better understanding of placebo analgesia requires a deeper theoretical comprehension of pain perception. We show that the question can be addressed by a Bayesian model of pain that considers the general properties of perception and those specific to pain and its modulation, as they emerged from electrophysiological and psychophysical experiments.

A first aspect is that pain perception depends not only on features of noxious stimuli, such as the severity of tissue damage, but is modulated by many psychological and cognitive factors, like motivational states, attention, emotions, expectation, memories and beliefs [11]. The effects of these factors are related to the complex role of pain, which is part of a response system that alerts and prepares the organism to face harmful situations [12]. Further, as for any sensory modality, pain is never a direct mapping from a physical space to a perceptive space [13, 14]. It only has to be effective for subsequent behavioral responses. For example, in particular circumstances no pain is felt in the presence of a lesion, while in other cases pain is perceived despite the absence of peripheral damage [15, 16]. At the same time, perception has to be reliable in spite of the uncertain information that sensory signals provide about the world, and this relates to probability theory [17, 18].

Probabilities may represent degrees of plausibility and probability rules are an effective way of reasoning under uncertainty. In particular, Bayes’ theorem was developed to calculate transposed conditional probabilities, for example the probability of an event (or a cause) *E* given some observations (or outcomes) *O*, Pr(*E*∣*O*), when we know its transposed conditional probability, that is the probability of *O* given *E*, Pr(*O*∣*E*). Bayes’ Theorem says that Pr(*E*∣*O*) is proportional to Pr(*O*∣*E*) (the likelihood), but also to Pr(*E*) (the prior probability, that is the probability of *E* beyond any knowledge about *O*, *e.g*. due to previous experience) and it is written:

$$\mathrm{Pr}\left(E|O\right)=\frac{\mathrm{Pr}(E)\times \mathrm{Pr}(O|E)}{\mathrm{Pr}(O)},$$ (1)

where Pr(*O*) is the probability of *O* regardless of *E*.

To deal with uncertainty we can use Bayesian decision analysis to identify, in the presence of uncertainty, the best choice: the one that minimizes the expected loss. We can accomplish this with a two stage process by first combining, together with past experience, multiple sources of information—Bayesian inference—and then using the resulting probabilities to weight the possible outcomes of alternatives—decision [19].

Interestingly, living organisms and their perceptive systems deal with uncertainty and face transposed conditional probability problems: they have to infer the features of activating stimuli (related to the state of the world) from nervous signals elicited by those stimuli, and come to a reliable perception (*i.e*., make an effective decision) in spite of the noisy and incomplete information that sensory signals provide about the world [20–22]. Considering Eq. 1 for the purpose of perceptual tasks, Pr(*E*∣*O*) provides the degree of plausibility of an event *E* (caused by a stimulus) given observations *O* (e.g., sensory signals) coded by nervous activity. Observations, and other information, may be collected from different sources. Indeed, experimental and theoretical work showed that perception is a multisensory task [23, 24], and support the hypothesis that, in perception, different pieces of information are near optimally combined in a Bayesian way [25, 26]. Models based on Bayesian decision theory [17, 27–29], developed mainly in research on vision, can account for relevant features of perception, including multi-modal integration [30, 31] and optic illusions [26, 32, 33], and have also been proposed for placebo analgesia [34–36].

In this study we focused on the placebo effect because it is one of the best examples of experimentally controllable modulation of pain experience, and has been extensively investigated in recent decades [2, 37]. In many studies about placebo, subjects were repeatedly stimulated under two conditions: treatment and no treatment, each paired with an unambiguous symbolic cue [8, 38, 39]. These experiments began with a conditioning stage, in which subjects experienced the analgesic efficacy of the treatment condition but, for ethical and practical reasons, in place of a real analgesic treatment stimuli cued for treatment were surreptitiously lowered. To test for the placebo effect, in a subsequent stage all the stimuli were delivered at the same intensity irrespective of the cue. Reconsidering this procedure in Bayesian terms, we assumed that the experience acquired through the training phase affects the prior probabilities and likelihoods for the stimulus perception. That is, at the end of the conditioning stage the system comes up with a probabilistic knowledge about the possible pain intensities in the specific context of the experimental session and learns, in terms of conditional probability distributions, the relationship between cues and pain intensities [34–36].

A main consideration in favor of the use of probability distributions comes from neurophysiological and psychophysical experiments, which showed a certain degree of variability, described by Gaussian distributions, in the relationship between stimulus intensity and both nociceptor activation and the perceived pain level [40–42].

Our aim was to develop a Bayesian framework which could describe and explain pain perception and its modulation. We can equate the task of perceiving pain to deciding which pain level should be perceived, given the information at hand. The decision should be related to the effect *E* of the stimulus on the tissue (*e.g*., the severity of the lesion), which cannot, however, be directly known. The nociceptive system must make an estimate through an inference process that it accomplishes using signals from nociceptors. As we argue here, the nociceptive signals are not the only source of information used to compute the inference: past experience and cognitive information also play a role.

An unexpected finding was that, besides the placebo effect, the Bayesian model made novel predictions about how previous experience can influence the perception of unexpected stimuli. This was a relevant aspect to test the model, and our experiments focused not only on the placebo effect but, more importantly, on a condition the model predicted and never reported.

## Materials and Methods

### Ethics Statement

This study was approved by the ethics committee of Azienda Ospedaliero Universitaria Santa Maria della Misericordia di Udine, Udine, Italy. All the experimental procedures were conducted in accordance with the policies and principles contained in the Declaration of Helsinki, and all subjects gave their informed consent in writing to take part in the study.

### Subjects

A total of 55 healthy human volunteers (mean age ± s.d.: 21.40 ± 1.03; 29 females) were recruited by advertising at the University of Udine (Italy) and randomly divided into two groups: Experiment 1 (*n* = 24); and Experiment 2 (*n* = 31). Prior to the experiment, each subject was informed in detail on the procedure to be used, and specifically that the study would asses a new protocol for Transcutaneous Electrical Nerve Stimulation (TENS—a technique able to induce analgesia by means of electric stimulation of afferent nerves).

### Experimental procedure

Subjects in both groups underwent a conditioning protocol, followed by a placebo test block (Experiment 1) or by a test block with no cue and a placebo test block (Experiment 2). We placed two electrodes for the sham analgesic treatment on the right or left ankle—chosen at random within the group—and a further two on the back of the same foot for painful electrical stimulation.

To select the intensities applied throughout all the experiment, we began the session by measuring the subject’s pain threshold and sensitivity according to the method of the limits [43]. During this stage, in agreement with the subject, we selected a high stimulus intensity; we then set a low intensity and, in Experiment 2 only, an intermediate one (at the midpoint between the high and low). Every subject should feel comfortable with the high intensity stimuli.

The experimental procedure was similar to that described by Colloca and Benedetti [39] and was subdivided into two stages (training and test). We told subjects they would receive painful electrical stimuli, and that TENS would be applied as an analgesic treatment paired with half of the stimuli. Subjects were instructed that the presence and absence of the analgesic treatment would be signaled on a PC screen, turning green or red respectively. The color choice and its association to the stimulus intensity was deliberate, as it is known that colors influence the placebo response (see the Limits and perspectives section in Results and discussion). In reality, for ethical and practical reasons, we applied no TENS, but surreptitiously lowered the painful stimulus paired with the green cue during the training stage in order to make the subjects experience the putative analgesia. On the contrary, during the test of the placebo effect, we always delivered high intensity stimuli, even when the green cue was presented. Subjects underwent a total of 3 (Experiment 1) or 4 (Experiment 2) blocks of stimulation and were not aware of any change of the stimulation protocol between blocks. Experiment 1 was aimed at testing the placebo effect and, in order to keep at minimum the possible extinction of the effect, we delivered 12 stimuli in each block. On the other hand, in Experiment 2 we were more focused on the responses to stimuli paired with no cue (third block), a condition, we predicted, with more dispersed rating. In this experiment we delivered 16 stimuli per block, with half of the stimuli of the third block used as reinforcers (see below).

The conditioning stimuli were delivered in the first two blocks, half at the high intensity, paired with red cues, and the others at the low intensity, paired with green cues, delivered in random order. After each stimulation, the subjects rated the perceived pain intensity through a Visual Analog Scale (VAS), presented on the PC screen after the visual cue. The aim of the conditioning was to make the subjects experience the effectiveness of the analgesic procedure and the magnitude of the analgesia.

The testing stage followed. As in training, visual cues were presented on the PC screen and subjects rated the perceived pain intensity through the VAS. The third block in Experiment 1 (first test block) and the fourth in Experiment 2 (second test block) tested for the placebo effect and were similar to the training session, however only the high intensity was used: stimuli were delivered randomly paired with the red or green cues, in equal proportion. In Experiment 2, the third block (first test block) aimed at testing the predictions of the theoretical model about subjects’ pain rating with no clue to the stimulus intensity. Unlike conditioning, during the first test block stimuli were paired with a neutral blue cue; three levels of intensity were chosen for the test: 4 high-intensity stimuli, 4 low-intensity stimuli and 8 intermediate-intensity stimuli (mid_blue_ stimuli), delivered in random order.

### Painful electrical stimuli

The electrical stimuli were square pulses delivered by a constant current high voltage stimulator (DS7A model, Digitimer Ltd, Welwyn Garden City, England), with intensities in the range 14–170 mA and a duration of 200 *μ*s. Two Ag/AgCl electrodes with foam and solid gel (ARBO, Germany; stimulation area = 2 cm^2^) were placed on the back of the foot. Stimuli were delivered at the end of either a red or green visual cue presented on a PC screen. We used an in-house MATLAB routine (MATLAB 7.1-R14, The MathWorks Inc., Natick, Massachusetts, USA) to present the visual cues and the VAS bar on the PC screen, and to collect the pain ratings of the subjects. Specifically, the subjects were instructed to use a computer mouse to change the length of a bar, according to the intensity of perceived pain. All the data were stored on a computer for the subsequent analyses.

### Modeling

In line with Bayesian Decision Theory we developed a two-stage model (full Bayesian decision model—fBD), which first estimates the severity of the tissue lesion (inference) and then chooses the optimal pain intensity to be perceived (decision).

#### Perceptual inference

Applying Bayes’ Theorem (Eq. 1) it is possible to update the prior probability given new observations (*O*). In a nociceptive context, *O* may be the nociceptor spiking activity (*N*), but may also include additional information. In particular, to model the placebo effect—hence the effect of expectation of analgesia and/or conditioning toward the context, shaped through the training stage—we explicitly considered the additional information provided through symbolic cues (*C*), *e.g*. red and green visual cues. To calculate the predictions of the model in the placebo test stage, we combined the expected effect of the stimulus (prior probability Pr(*E*)) with sensory inputs (nociceptor activity) and contextual information (through the paired cue) which both enter the Bayes’ rule through likelihood functions so that, if *N* and *C* are independent, Pr(*O*∣*E*) = Pr(*N, C*∣*E*) = Pr(*N*∣*E*) × Pr(*C*∣*E*). Using the Bayes’ rule we calculated the posterior probability distribution of *E*, Pr(*E*∣*O*), considering the nociceptor spiking activity either alone (*O* = *N*):

Pr(E∣N)∝Pr(E)×Pr(N∣E) (2)

or jointly with the cue (*O* = *N, C*):

$$\begin{array}{cc} \mathrm{Pr}(E|N,C) & \propto \mathrm{Pr}(E)\times \mathrm{Pr}(C|E)\times \mathrm{Pr}(N|E)\\ =\mathrm{Pr}(E|C)\times \mathrm{Pr}(N|E) \end{array}$$ (3)

where we omitted the denominator in Eq. 1 and replaced the equality with proportionality, the denominator being a constant needed to make the posterior probabilities sum to one (see the Placebo section in Result and discussion for further considerations on Eq. 3).

We assumed that the prior probability distribution of *E* (the effect of the stimulus on the tissue) after conditioning is a mixture of distributions:

$$\mathrm{Pr}\left(E\right)={\mathrm{Norm}}_{1}\times w/2+{\mathrm{Norm}}_{2}\times w/2+\mathrm{Unif}\times \left(1-w\right),$$ (4)

that is, two normal distributions—one for each stimulus used in conditioning—and a uniform one whose contribution (1—*w*) is smaller the stronger the effectiveness of conditioning and the contribution of expectation (*w*). We assumed that the two normal distributions have equal weights (*w*/2) when the two stimuli are delivered in equal proportions during training, and that they had the same dispersion and a mean proportional to the stimulus intensity used in conditioning:

$${\mathrm{Norm}}_{i}∼N\left(\mu \propto s_{i},\sigma \right),$$ (5)

with *s*_*i*_ and *μ* in the range 0–100 (arbitrary units). We chose the value *σ* fitting this parameter on the VAS ratings obtained in Experiment 1. Instead, any value of *w* may be used to explore the behavior of the model. However, to predict single subject ratings we needed to estimate the effectiveness of conditioning and the contribution of expectation (parameter *w* in Eq. 4) for each subject. In that case we fitted *w* on single subjects’ data.

We also assumed that, on the basis of electrophysiological data [41, 42], the response of nociceptors to stimuli—expressed by the likelihood function Pr(*N*∣*E*)—has a normal distribution and that it is not affected by the experimental procedure, being a relation between the effect of the stimulus on the tissue and the evoked nociceptor activity. For any *e*_*j*_ ∈ *E* we attributed it as follows:

$$\mathrm{Pr}\left(N|e_{j},X\right)∼N\left(\mu \propto e_{j},s.d.=0.75\right),$$ (6)

where the values for *μ* and *σ* are expressed in arbitrary units but broadly consistent with somatosensory neuronal responses [41, 42].

#### Decision and pain rating

Inference, formalized by Eqs. 2 and 3, provides probability distributions, but when we feel pain we do not perceive a spectrum of possible pain intensities, nor do we have a probabilistic representation of them: we perceive a specific pain intensity. We can achieve this *collapsing* the whole distribution to a point estimate—a single representative value; such a synthesis is operated through a decision rule. Indeed, in Bayesian decision theory the inference outcome is the basis of decision, which is performed by applying a cost function to the results of the inference and then choosing according to a rule [19, 44, 45]. In nociception this means that, even if everything else is the same, pain may change with different cost functions or decision rules. This also makes sense in behavioral terms, as different tasks or situations may require different responses to equally severe lesions [15, 16]. Assuming that the cost function is uniform, *i.e*. each unfit decision entails the same cost, the Bayesian decision criterion that minimize the expected loss is to choose the most probable intensity. To make a decision about *E*, that is to estimate the magnitude of *E* (*Ê*), we used the posterior distribution Pr(*E*∣*N, C*) (Eq. 3) and chose the *e*_*j*_ ∈ *E* with the maximum posterior probability [46], that is:

$$\hat{E}=e_{j}\;\text{if}\;\text{Pr}\left(e_{j}|N,C\right)>\mathrm{Pr}\left(e_{k}|N,C\right)\;\text{for}\;\text{all}\;k\neq j.$$ (7)

In a natural environment this is not necessarily true. However, in the experimental set of the placebo conditioning, a uniform cost function is likely to be a good approximation. In fact, subjects only have to rate their pain with maximal accuracy, with no advantage to one choice over the other. Moreover, subjects know and agree with the stimulus intensity and feel comfortable with it, as it is selected with them at the beginning of the experimental session according to their susceptibility and pain acceptance. Under such assumptions, which we accepted for the following modeling, the perceived pain intensity is directly proportional to the most probable effect on the tissue.

Finally, our aim was to model the pain rating (*R*) as a probabilistic function of the stimulus intensity (*S*) and of the expectation driven by the context (signaled by the cue): Pr(*R*∣*S, C*). In fact, as observers, we neither know the actual effect on the tissue (*E*) eliciting transduction at the nociceptor level nor the actual perception of the subject. Although decision generates a point estimate, multiple ratings of pain induced by the same stimulus intensity follow a Gaussian distribution [40]. To esteem subjects’ rating given stimuli and cue we combined *Ê* with the probability distributions of two sources of uncertainty, which are the variability associated to coding *Ê* into a score and the variability of *N* given a stimulus *S*:

$$\mathrm{Pr}\left(R|S,C\right)=\mathrm{Pr}\left(R|\hat{E}\right)\times \mathrm{Pr}\left(N|S\right),$$ (8)

where we considered Pr(*R*∣*Ê*) and Pr(*N*∣*S*) normally distributed [40–42]. According to electrophysiological studies [41, 42] we set the standard deviation of the distribution of Pr(*N*∣*S*) = 0.75 (and the mean proportional to the stimulus intensity), while we estimated the standard deviation of Pr(*R*∣*Ê*) fitting the model to individual subjects’ data.

### Data analysis

We used the ‘*R*’ statistical environment (R Foundation for Statistical Computing, Vienna, Austria) [47] to implement the Bayesian model, for statistical computing and to generate graphics.

To allow comparisons between subjects, all the VAS scores were scaled relative to the mean response to the high stimuli intra-condition; in the placebo condition the scores were scaled relative to the mean VAS rating of the high stimuli paired with the red cue.

Since the model predicts that posterior distributions are not Gaussian, we performed statistical comparisons of the experimental data by means of the Wilcoxon signed-rank test and the Mann-Whitney rank-sum test (accepting a probability value of 0.05 (one tail) as the level of statistical significance in all the tests performed).

We clustered the data with the Hartigan and Wong *K-means* method [48]. As a measure of clustering we chose the distance between the centers of the clusters and a separation index, calculated as the ratio of the between clusters sum of squares and the total sum of squares, where the between clusters sum of squares was calculated as the difference between the total sum of squares and the sum of the within clusters sum of squares.

We estimated correlations by means of the Pearson’s product-moment correlation coefficient (accepting a probability value of 0.05 (one tail) as the level of statistical significance in all the tests performed).

#### Model comparison

To test how the responses to mid_blue_ stimuli (Experiment 2) were distributed and to compare our model with possible alternatives we followed a Bayesian approach. We assigned equal priors for the competing hypotheses or models and computed posterior probabilities; we then compared the alternatives by means of the Bayes Factor [49].

To test if, as the model predicted (see Results and Discussion), after conditioning the subjects had a greater probability to feel low or high pain in response to mid_blue_ stimuli, we tested the hypothesis of bimodal distribution against the hypothesis of unimodal distribution. First we used the data (VAS scores for intermediate intensity stimuli in the third block of Experiment 2) to define the two alternative distributions, then we calculated the posterior probabilities, and the Bayes Factor, for the two hypotheses given the data. The unimodal distribution was a Gaussian one with mean and standard deviation equaled to those of the VAS scores considered all together. The bimodal distribution was a mixture of two Gaussians whose means and SDs equaled those of the two clusters identified by the Hartigan and Wong algorithm. A further comparison, in which the two distributions were built from a different data set, is provided by the model comparison described below.

For model comparison we used data of Experiment 2, scaled intra-block (see above). First, we considered each subject’s high and low stimuli scores (from the two conditioning blocks) as the reference intensities for modeling: the mean VAS rating for high stimuli and that for low stimuli were set as the means (parameter *μ*) for Norm_1_ and Norm_2_ in Eqs. 4 and 5, while their standard deviations were used to express the variability of Pr(*R*∣*Ê*) in Eq. 8; then, we fitted the model on a first set of data (the placebo data collected in the fourth block) and so quantified the effectiveness of conditioning and expectation—parameter *w* in Eq. 4—for each subject; finally, we compared the models using a second set of data (the VAS scores obtained in the third block with the intermediate intensity stimuli and no-cue). Estimating the parameters of the model on one data set and comparing the model by means of a different one avoided the use of the same data twice, and the need of correcting strategies (such as the Bayesian Information Criterion, BIC, or of the Deviance Information Criterion, DIC). This approach also overcomes a drawback which, as stated by Maloney and Zhang [29], is common in Bayesian modeling of perception: “*After the data are collected it is not very difficult to develop a Bayesian model that accounts for it. Indeed, almost many applications of Bayesian methods to perception and action are* post hoc *fitting exercises. If Bayesian models are to be judged useful, then, they must also permit prediction of experimental outcomes, quantitatively as well as qualitatively*”.

We compared the full Bayesian decision model with two alternatives: a *no-learn* and a *simple* Bayesian model. In the *no-learn model* the conditioning stage does not affect pain perception. This model predicts that, when a subject receives mid_blue_ stimuli, pain ratings follow a unimodal distribution, with the mean at midpoint between the pain levels reported during conditioning (with the low and high stimuli). The *simple Bayesian model* integrates, in a Bayesian way, the expectation of the pain intensity (prior) with the sensory input due to the stimulus (likelihood). In the case of mid_blue_ stimuli this model integrates the expectation of high and low pain intensities experienced in the conditioning stage with the sensory input due to intermediate stimuli. Like the one we propose, the simple Bayesian model predicts a bimodal distribution of pain ratings.

## Results and Discussion

In the following, we first deal with parameter selection for the model and then with the evidence that, through model comparison, supports the full Bayesian decision model (fBD). Only after that we deal with the predictions of the model and the insights it provides into pain perception and its modulation.

### Parameter estimation

We built the full Bayesian decision model (fBD) with the goal of optimally integrating past experience and incoming information about the stimulus (provided by nociceptor activity) and about the surrounding context (provided by the cue). The parameters of the model were defined to reflect the knowledge available to the system either before or at the end of the conditioning phase, and to mirror neurophysiological and psychophysical evidences. We fitted the model to Experiment 1 data and found that the standard deviation of the effects induced by the stimulus (parameter *σ* in Eq. 5) was 5.7 *±* 3.1 (mean ± s.d.).

**Figure 1:**
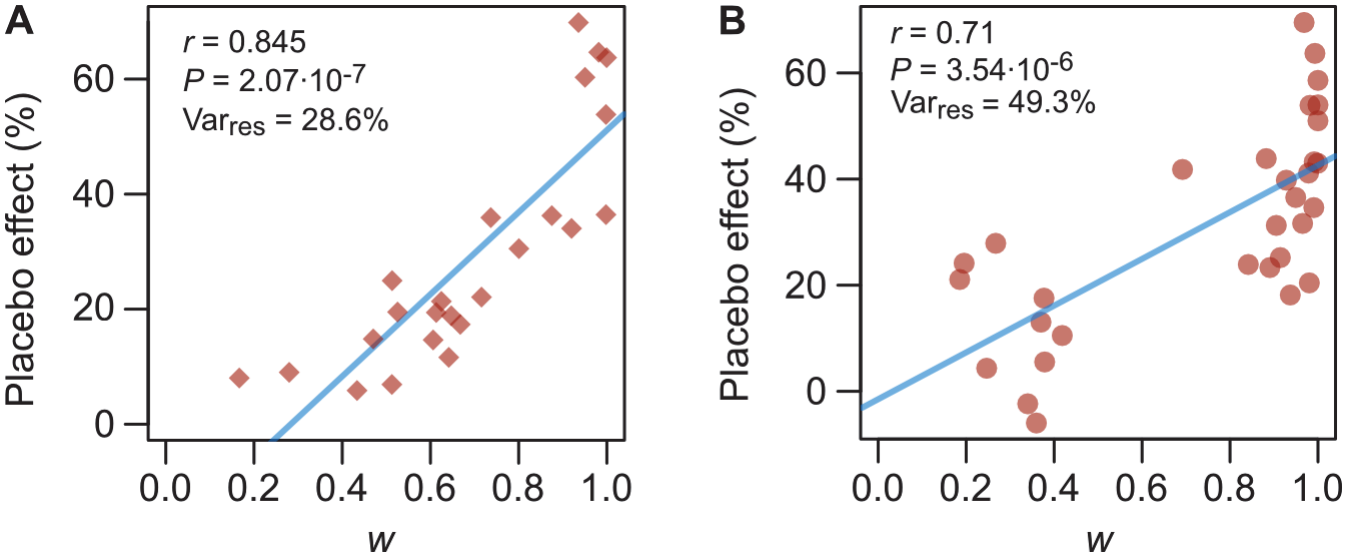
Correlation between subjects’ expectation of analgesia and placebo analgesia ratings. The expectation of analgesia / effectiveness of conditioning (parameter *w*) was estimated fitting the model on individual subjects’ placebo data. For Experiment 1 (**A**) we fitted also, on subjects’ data, the dispersion of the prior distribution of the effect of the stimuli (Eq. 5), while for Experiment 2 (**B**) the dispersion entered in the model was the one estimated on Experiment 1 data. The plots show a positive correlation between expectation/conditioning and the size of the placebo effect. *r*: Pearson’s product moment correlation coefficient, tested for positive correlation. Var_res_ = residual variance. (*n* = 24, Experiment 1; *n* = 31, Experiment 2).

We also estimated, separately on placebo scores of Experiment 1 and 2, the effectiveness of conditioning and expectation (parameter *w* in Eq. 4) in each subject. As expected, *w* positively correlated with the magnitude of the placebo effect (Fig. 1; Pearsons’ correlation coefficient *r* = 0.84, *P* = 2.07 × 10^−7^ (Experiment 1); *r* = 0.71, *P* = 3.54×10^−6^ (Experiment 2)), however other sources of individual variability still remain, as *w* can explain a relevant part, but not all, of the variance of the placebo effect (in Experiment 1, where we fitted the parameters *σ* (Eq. 5) and *w* on single subjects’ data, the residual variance was 28.6%; in Experiment 2, where for modeling we used the parameter *σ* calculated from Experiment 1 data, the residual variance was 49.3%).

### Model comparison

**Figure 2:**
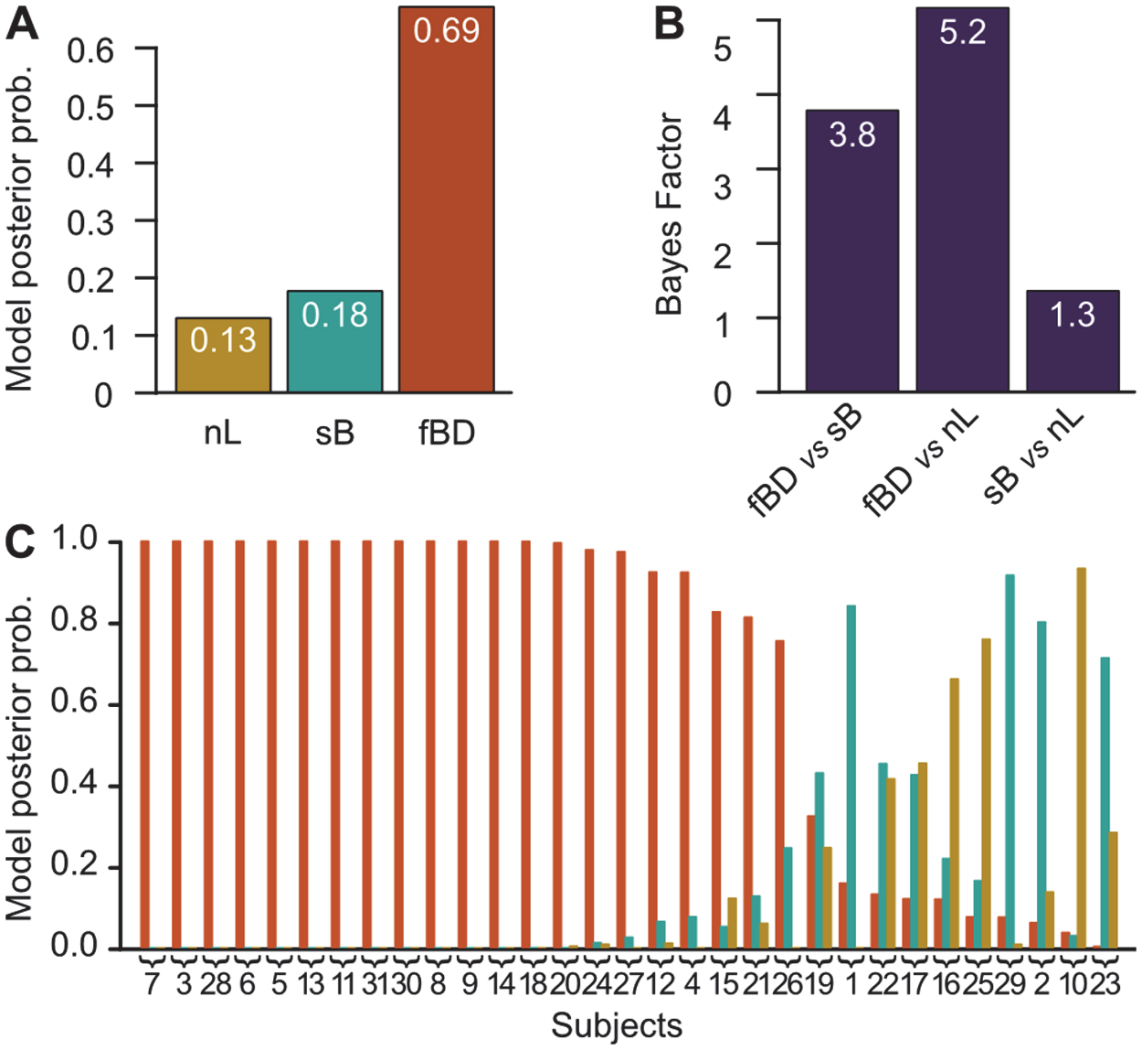
Model comparison. (**A**) Posterior probability and (**B**) Bayes factors for the three models we compared, showing a substantial evidence in favour of the full Bayesian decision model we developed. (**C**) Posterior probability of the three models in each subject (*n* = 31), showing that in most of the subjects the data support the fBD model. Models are identified by the same colors in (A) and (C). nL = no learn model; sB = simple Bayesian model; fBD = full Bayesian decision model.

We compared the fBD model with two alternative ones (see Methods) calculating the posterior probabilities for the models given the data collected in the third block of Experiment 2 (that is pain induced, after the conditioning stage, by mid_blue_ stimuli; see the No cue *section* below for a description of the outcomes). In the comparison, the data supported the fBD model: it had the greatest posterior probability and, according to Jeffreys’ Bayes Factor scale of evidence [49], it is favored by substantial evidence (Fig. 2).

Note that in choosing the alternative models for comparison we aligned with the principle of avoiding *post hoc* fitting of the data: although a number of models may be conceived to fit the data, the two we chose are the most straightforward, the others being built with the principal aim to fit the data but with poor theoretical justification. Also, such an exercise would be a complex one if aimed at fitting both the placebo response and the rating of the pain induced by the mid_blue_ stimuli. For example, we discarded the model recently proposed by Yoshida et al. [36] as we could not match their findings. This is likely due to the different protocols: the uncertainty in conditioning, experienced by a subject (which we can model), carries a different meaning than the uncertainty in expected pain due to ratings reported to the subject by other people (the condition tested by Yoshida et al.). A second reason is that attempting and tuning their model to our placebo data, resulted in no usable predictions for the mid_blue_ condition.

The two other models included in the comparison were (1) a model which assumed there was no learning in conditioning, which performed poorly compared with the others, and (2) a model which uses data from the conditioning stage to infer a Bayesian (posterior) estimate of pain in the placebo condition and with mid_blue_ stimuli, which performed slightly better (Fig. 2).

When looking at single subject’s responses to mid_blue_ stimuli (after the conditioning stage), the fBD model agreed with pain experienced by most subjects (Fig. 2C).

### Model predictions

**Figure 3:**
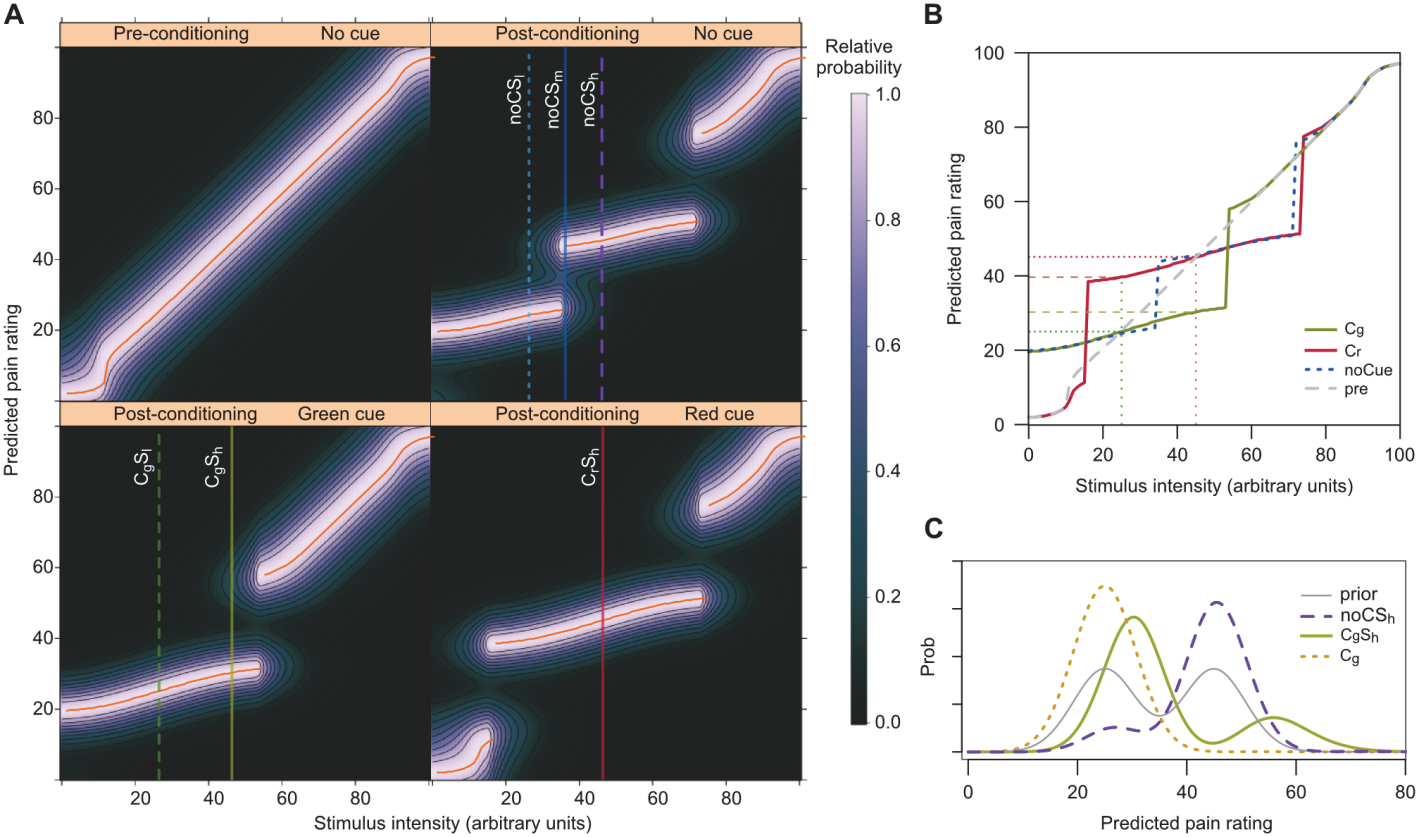
Model predictions of pain rating. Probability distributions before (**A**, **B**) and after (**A**, **B**, **C**) conditioning. (**A**) Posterior probability distributions of pain rating given the stimulus intensity. The color scale codes for relative probabilities (scaled so the maximum equals 1). Orange curves indicate maxima (most probable pain rating), also reported in (B). Vertical lines highlight some of the distributions shown, with same colors and line types, in (C) and in Figs. 4C and 5A. (**B**) Most probable rating given a stimulus, for each possible stimulus: before training (pre), and after training. Values after training are shown for stimuli paired with a cue (C_g_: green cue; C_r_: red cue) or not (noCue). Horizontal lines indicate the estimated pain rating for high stimuli paired with red (red dotted line, overt no-treatment) and green (green dashed line, placebo condition) cues, and for low stimuli paired with green (green dotted line, overt treatment) and red (brown dashed line, nocebo condition) cues. (**C**) Prior probability distribution (prior), and posterior probability distributions conditioned on the high stimulus (S_h_), on the green cue (C_g_), and on both the high stimulus and the green cue together (C_g_S_h_, placebo).

The full set of posterior probability distributions of pain rating, calculated for any stimulus intensity and with *w* (the effectiveness of conditioning/expectation, see Eq. 4) set to 0.9, is shown in Fig. 3. The first thing to note is that the relationship between the stimulus intensity and the most probable pain rating changes significantly as a consequence of the conditioning stage (Fig. 3A and B). Prior to conditioning this relationship is almost linear (as in the case of thermal pain [50]), but after conditioning it becomes non linear, in a way specific to the paired cue or its absence (Fig. 3A and B).

It is important to note that, for model predictions, we calculated the effect of the first stimulus after conditioning; that is, we did not model the possible extinction of the response. A further development of the model would also include the ongoing learning of the system after each stimulus. This will provide insights both in the shaping of the posterior probability distribution during conditioning and in the evolving of the response, stimulus after stimulus, during the testing stage.

#### Placebo

**Figure 4:**
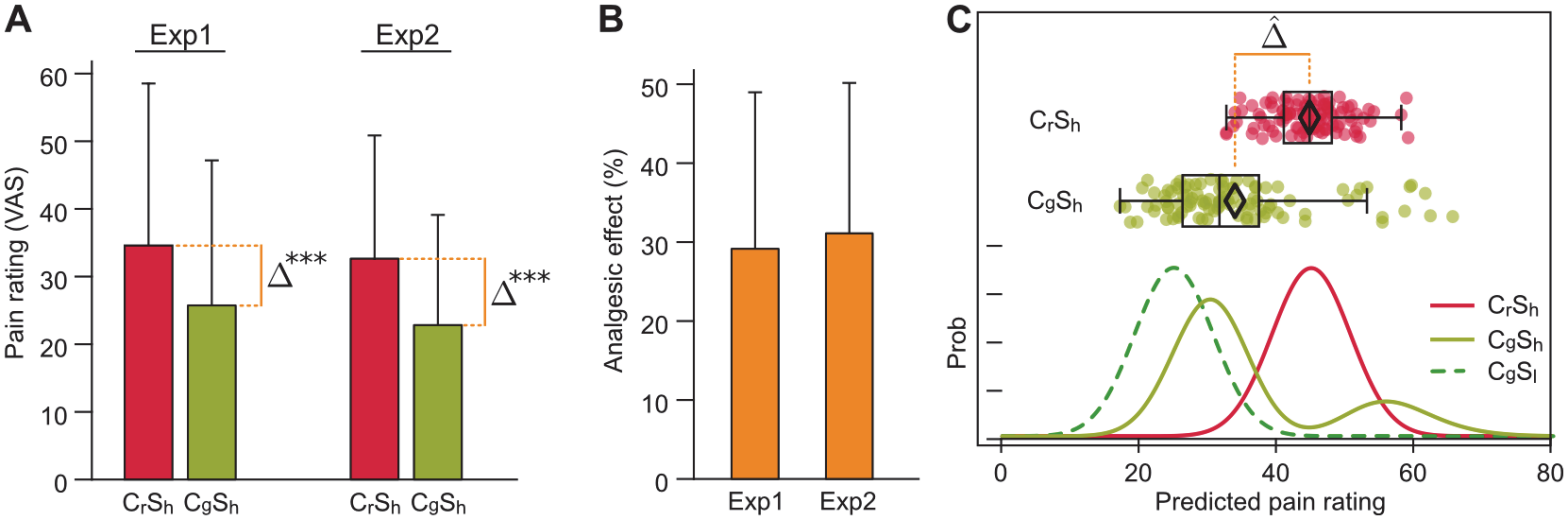
Placebo effect. (**A**, **B**) Experimental placebo effect after conditioning, observed in the two samples of participants recruited for Experiment 1 (*left columns*) and Experiment 2 (*right columns*). (**A**) Mean pain scoring, rated through a visual analogical scale (VAS), for high electrical stimuli paired with red (C_r_S_h_, overt no-treatment) or green (C_g_S_h_, placebo condition) cues. (**B**) Mean placebo analgesic effect, as percent difference relative to pain rating with overt no-treatment. (**C**) Model predictions: box-plot and 100 random draws (*top*) from the posterior probability distributions (*bottom*) for high stimuli paired with red (C_r_S_h_, no-treatment) or green (C_g_S_h_, placebo) cues. The posterior probability distribution for the low stimulus paired with the green cue is also plotted (C_g_S_l_, overt treatment). Δ = observed placebo analgesia; $\hat{\Delta }$ = predicted placebo analgesia (difference between the means (◊) of the no-treatment and placebo samples). ***P < 0.001 (Experiment 1: *P* = 5.96 × 10^−8^, *n* = 24; Experiment 2: *P* = 2.00 × 10^−8^, *n* = 31; (one tail Wilcoxon signed-rank test); all error bars represent s.d.

When a stimulus is paired with a deceptive cue the model predicts pain ratings that correspond to the placebo effect (Figs. 3 and 4)—or to its reversal, the nocebo effect [51–53] (Fig. 3B). As an example, in Fig. 4 we report a comparison between the model outcomes and actual placebo effect from the two experiments we performed on healthy human volunteers.

The fBD model shows that when independent channels carry congruent information, they have the potential to make the esteem more accurate: notice the narrower distribution in Fig. 3A. But in the placebo condition, when the stimulation is manipulated so that information is deceptively discordant, the green cue, which retains its learned meaning, biases the esteem toward the lower stimulus. In fact, notice that the knowledge about the cue affects the shape and peak position of the probability distributions (Fig. 3A and C, and Fig. 4C) and, as a consequence, the perceived pain to the same stimulus (Fig. 3B and 4C).

Studies on the placebo effect found that it can be due to expectation or conditioning [1–4]. Hence, in models of pain inference cues should be considered, respectively, as contextual information shaping expectation and so prior probability, or as additional information which, as sensory information, is integrated through a likelihood function. Although biologically different, in Bayesian inference these two procedures produce the same result and, in Eq. 3, we wrote the two equivalent forms. In Bayesian inference, in fact, the posterior probability may be calculated combining each piece of information in subsequent steps, where the posterior probability which results from one step provides the prior probability for the next step, or combining multiple pieces of information at the same time, through their respective likelihood functions.

#### No cue

**Figure 5:**
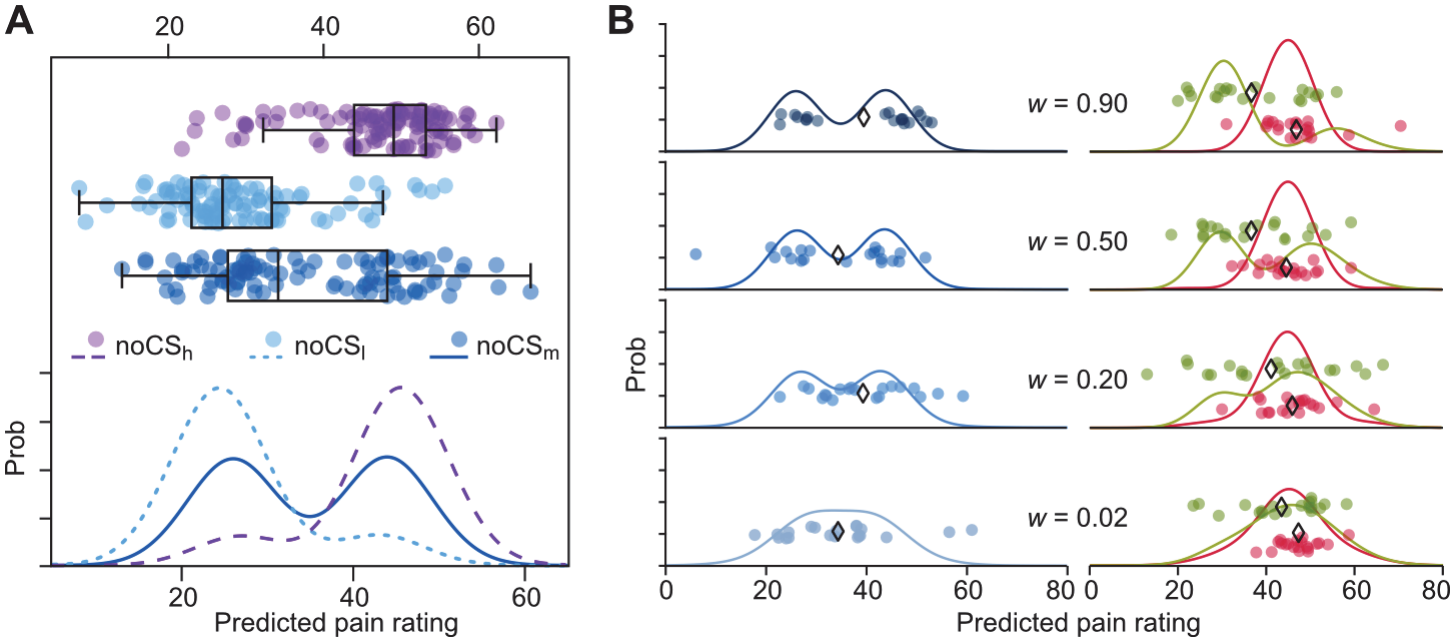
Model predictions of pain rating with and without cues, after conditioning. (**A**) 100 random draws (*top*) from posterior probability distributions (*bottom*) for high (noCS_h_), low (noCS_l_) and intermediate (noCS_m_) stimulus intensities, paired with no cue. (**B**) Probability distributions of pain rating obtained with different effectiveness of conditioning (*w* = weight factor attributed to conditioning), and 20 random draws from each probability distribution. Predictions for intermediate stimuli paired with no cue (mid_blue_ stimuli, *left*) are displayed aside those for high stimuli (*right*) paired with green (green circles and curves, placebo condition) and red (red circles and curves, overt no-treatment condition) cues. ◊ = means of each sample.

**Figure 6:**
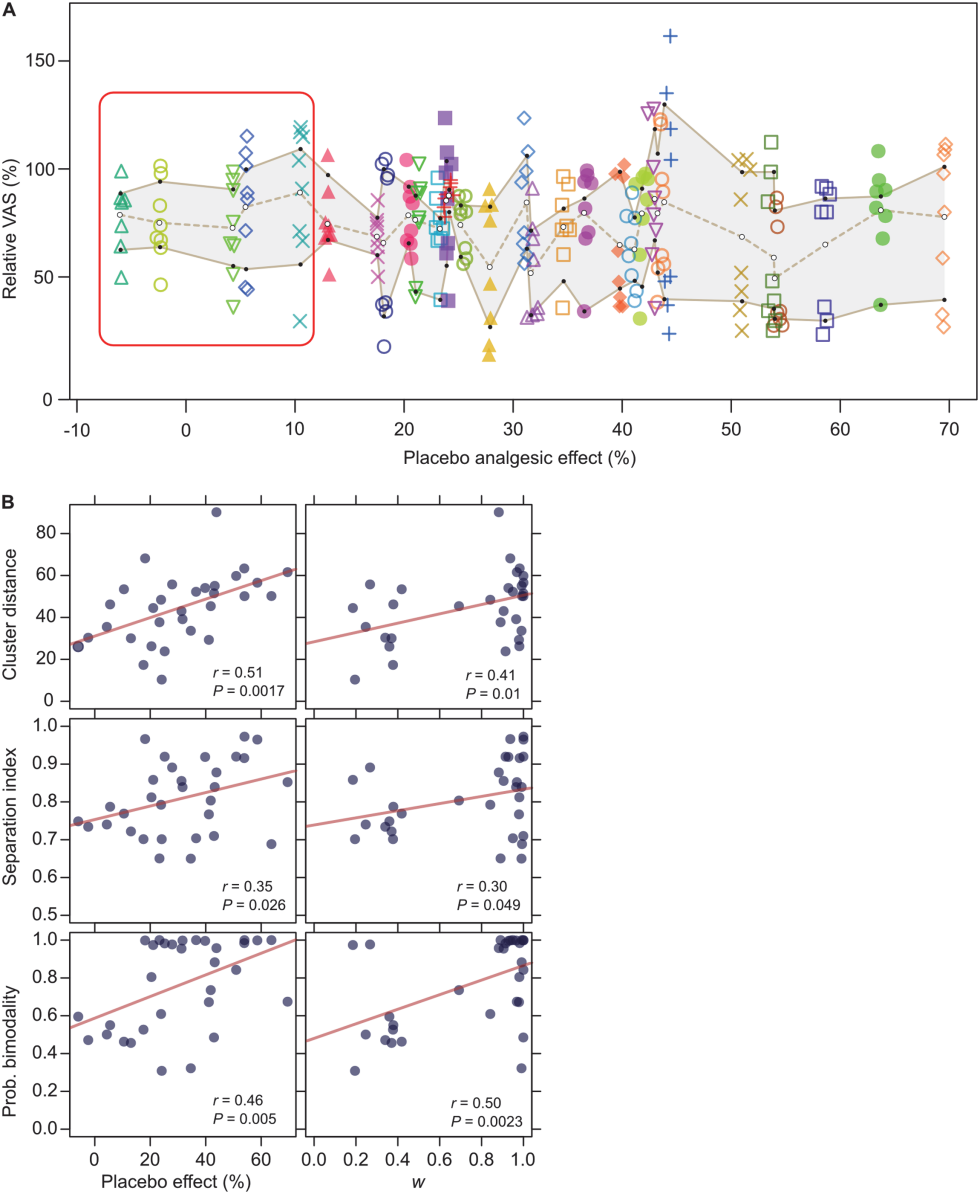
Ratings of pain induced by intermediate intensity electrical stimuli paired with no cue. (**A**) 31 subjects’ pain rating (*y* axis) scaled, for each subject, to the mean of pain ratings for high intensity stimuli in the same stimulation block); subjects are ordered according to the magnitude of the placebo effect (*x* axis, the magnitude is relative to the mean of pain ratings for high intensity stimuli paired with red cues). Each subject rated 8 stimuli and is represented with a different color and symbol. The square box delimits subjects with no significant placebo effect (tested at *P* < 0.05; *n* = 8; one tail Mann-Whitney rank-sum test). (**B**) Correlation between individual clustering measures of pain rating (cluster distance, *first column*; cluster separation index, *second column*; probability that the data followed a bimodal distribution, *third column*) and the magnitude of the placebo effect (*first row*); or the expectation of analgesia (parameter *w*) estimated by the model on placebo data (*second row*). Cluster analysis: *K-means* method for 2 clusters; test of bimodal distribution *vs* unimodal: Bayesian hypothesis comparison; correlation analysis: Pearson’s product moment correlation coefficient, tested for positive correlation. (*n* = 31). (See also Table 1).

The main findings of this study concern not only the placebo effect but a wider range of effects also due to past experience. To test these predictions, in our study we focused on the pain rating for stimuli of intensity at midpoint between those used in conditioning and delivered in the absence of a visual cue (mid_blue_ stimuli; Fig. 3A, upper right panel; Fig. 3B, blue dotted curve; and Fig. 5). The fBD model predicts that, with a uniform loss function, the probabilities of the perceived pain would follow a bimodal distribution, with pain ratings more likely to be clustered around the two peaks of highest probability (Fig. 5A), and that the degree of clustering is positively correlated with the effectiveness of conditioning/expectation (Fig. 5B). These outcomes were unexpected, but the experimental results supported them. In fact, the subjects’ responses to intermediate-intensity stimuli were mostly clustered toward the two levels perceived within the conditioning stage, as the model predicts, and only in some cases to the midpoint, as one would otherwise expect (Fig. 6A).

The first formal evidence that the ratings followed a bimodal distributions is provided by the model comparison described above: the bimodal models were more probable then the unimodal one (Fig. 2).

For a further comparison between unimodality and bimodality we used Bayesian hypothesis testing. Again, the hypothesis of bimodality resulted to be the most probable, favoured by substantial evidence (posterior probability = 0.77; Bayes Factor = 3.85).

Finally, we tested the predicted relationship between clustering and subjects’ expectation/conditioning (Fig. 5B). In agreement with the model, we found that the greater the placebo effect in a subject, the more evident and pronounced the clustering was (Fig. 6), suggesting that the heterogeneity of results among subjects may be due to differences in the effectiveness of the conditioning procedure. Indeed, both the size of the placebo effect and the subjects’ expectancy/conditioning (parameter *w*, estimated by the model on the placebo data) positively correlated with the measures of clustering (the distance of the cluster centers and the index of cluster separation) and with the probability that the data followed a bimodal distribution (Fig. 6B and Table 1).

**Table 1:** Cluster correlation.

	**Cluster distance**	**Separation index**	**Prob. bimodality**
Placebo analgesia size	*r* = 0.51 (*P* = 0.0017)	*r* = 0.35 (*P* = 0.026)	*r* = 0.46 (*P* = 0.0050)
Expectation (*w*)	*r* = 0.41 (*P* = 0.010)	*r* = 0.30 (*P* = 0.049)	*r* = 0.50 (*P* = 0.0023)

Correlation between individual clustering measures of pain rating and the magnitude of the placebo analgesia, or the expectation of analgesia (parameter *w*) estimated by the model on placebo data. Subjects’ pain ratings were clustered with the *K-means* method for 2 clusters, whereas bimodal *vs* unimodal distribution was assessed with Bayesian hypothesis comparison. Cluster distance: distance of the cluster centers; Separation index: cluster separation index; Prob. bimodality: probability that the data follow a bimodal distribution; *r*: Pearson’s product moment correlation coefficient, tested for positive correlation. (*n* = 31). (See also Fig. 6B).

We know that past experience affects both perception [27, 54, 55] and the placebo effect [39, 56]. Our findings show that experience shapes not only the relevance of additional information—as in the case of the placebo effect, in which elements of the context (here the cues) become more likely associated with specific pain intensities—but also the expected pain levels independent of the cue. In Bayesian terms, the former means that past experience changes the likelihoods related to additional informative symbolic cues, while the latter means that experiencing which pain levels are possible within the experimental session affects also the priors for perception in that context (Fig. 3A and C).

### Limits and perspectives

The Bayesian decision model we developed comprises three key elements: the prior probability, which conveys previous experiences and expectancy (e.g. through information derived from the context); the likelihood function, which implements the sensory inputs and also information from multiple sources (whether sensory, cognitive or psychological); and the decision process, which eventually determines if and to what extent pain is perceived.

In this work we mainly investigated the inference process, however decision making is a complex and essential part of perception, which involves many neural subsystems, including sensory and cognitive networks [57], and is likely to be of great relevance in describing many significant conditions of pain modulation, such as the analgesia observed in life-threatening situations, or during high motivation states: perception involves the evaluation not only of the severity of the lesion, but also of the consequences of the behavioral choices based on that perception. Although our model includes a cost function (for simplicity we assumed a uniform one) and although we repeatedly discussed its role and the effects of different cost functions on pain perception, we have not explored the decision side of the model. For a deeper understanding of pain and its modulation, further studies have to consider the effects of additional variables (*e.g*., related to emotional states, attention, motivation, etc.) both on inference (through likelihoods) and on the decision process. A work which focused on the relevance of decision process in pain is the recent study [58] in which Wiech et al. stated that “*decision-making is pivotal to the modulation of pain*” and that “*cognitive pain modulation can also be rooted in altered decision-making*”. We agree with those assertions but not with their conclusions: their results did not necessarily demonstrate that prior information *directly* biased decision-making. They found that prior knowledge altered decision-making (with effects on accuracy and response time) but not the sensory processing. In our framework expectation affects inference and, through that, decision. When we implemented in our model the experimental design of Wiech et al. (data not shown) we found that, due to the effect of expectation on inference, decision-making faces a greater or smaller uncertainty about the effect of the stimulus (wider or narrower posterior distribution), with consequences (on accuracy and, possibly, response time) consistent with their data. However, the insights provided by the two models are so different that a detailed comparison is not straightforward and would require further work. For example, a point to consider is if the bias on classification due to prior knowledge found by Wiech et al. is part of pain modulation or, instead, it affects a subsequent cognitive process.

Note that the fBD model does not specify the regions of the nervous system where the probabilistic integration of information and the decision making processes take place, nor the neuronal networks or molecular systems involved. In fact the model is concerned with the computational strategies and not with the neurobiological implementation of those strategies. However, recent studies have shown that signal integration at the neuronal level may behave according to Bayesian decision theory [59–61], and identified the neural substrates of Bayesian integration in some multisensory perceptual tasks [31, 62].

The conditioning protocol deserves some considerations. In this work we often refer to *conditioning* in a broad sense, meaning the processes which results in the placebo response and due to different elements of the experimental procedure. These elements include the implicit conditioning and the explicit learning obtained through the training stage, but also the changes induced by others factors, such as the operator’s description of the expected analgesic effect of the treatment. Indeed, to maximize the expectancy driven by the cue we relied on the contributions of different strategies. (1) Verbal instructions: we explained to the subjects the analgesic effect of the treatment and the association of red and green cues with intense and mild pain, as expectation of benefit is a component in the placebo response [63, 64]. (2) Non verbal suggestion: different studies have shown that context, environment, operator’s behavior and attitude, protocol and treatment procedures not only act to elicit a conditioned response [63] but also affect trust and credibility, which have a relevant role in the placebo effect [65, 66]. (3) Learning: before testing, the subjects underwent a conditioning stage, in which they experienced the association of the cues with pain intensity; in fact many studies demonstrated that learning strongly enhances the placebo effect [6, 39, 56, 63]. (4) Intrinsic expectancy: we paired red and green with high and low intensities respectively because the color may affect the placebo response (as it has been demonstrated for the color of medication [67, 68]): red is linked to danger, hazard and harm [69] so that red may carry an intrinsic expectancy of higher pain.

A caution about the pain felt by the subjects: the large electrodes we used (2 cm^2^) may activate many non-nociceptive fibers. Although previous research on pain and placebo analgesia have used such electrodes (e.g. Colloca and Benedetti [39]), and although our subjects described the stimuli as painful, we can not rule out the possibility that, at least in part, the placebo effect was not on pain but on discomfort.

As shown in Fig. 6A (*x* axis), subjects’ responses to placebo varied from almost 70% of analgesia to no response at all. Different studies investigated why healthy subjects in the same conditions may have different responses. What emerged is that different variables (related to cognitive and emotional factors and to personality traits) may account for the differences, and also that the same individual may show a placebo effect in some circumstances or with particular procedures and not with others [70–72]. We found that the individual expectation/conditioning (captured by the parameter *w*) accounted for a relevant part of the variability (50 to 70%), but a significant amount remained unexplained. According to our framework, differences between subjects may happen not only because subjects may have different priors (as in the case of expectation due to past experience), but also because they may consider different likelihood functions (that is, they may integrate different pieces of information and/or may give a different weight to the same information), or different cost functions. The model we explored suggests that the effects originating at these different levels may leave their own signature, which might be detected by proper psychophysical tests. For example, a weak expectation of analgesia as well as the hyperalgesia due to anxiety [73–75] may both result in a weak placebo response, but we expect they will differ in other responses and that specific tests may be conceived to detect them. An example is the response to mid_blue_ stimuli: as we have shown, in this condition a weak expectation corresponds to less or no clustering of the pain ratings—which would tend toward an intermediate level. On the other hand, with high expectation but a cost function which favors hyperalgesia we would expect either two clusters, but pushed to the right, or only one cluster corresponding to the rightmost peak of the distribution.

We believe that using a theoretical model—and the predictions it makes—to investigate and manipulate psychophysical behaviors may help to explore the interplay of factors modulating pain and to design tests to identify them not only in experimental protocols but even in clinical situations. Indeed we think that the modular structure of the model provides a conceptual framework which can also guide the investigation of pathological pain, as the underlying altered states may be due to specific modifications at one or more of the levels outlined. To adapt the model to pathological conditions may be challenging and will require further investigations, but its framework may already provide some hints. For instance, deficits in neuronal transmission (e.g., in injured nerves) or plastic circuitry changes can be considered when calculating the likelihood function; altered networks may also be modeled through the prior probability; the role of cognitive factors can be reflected by the prior probability or adding further likelihood functions; behavioral and motivational aspects of pain could be considered in the cost function and/or the decision rule.

## Conclusions

The model we developed shows how the placebo effect results from the evaluation and integration of nociceptive stimuli with context information, and how the relevance of the context (through expectation/conditioning) comes, at least in part, from past experience. The process of information integration would be at the very base of pain perception, and would lead to the placebo effect and to other phenomena such as those predicted by the fBD model.

Overall, our findings support the hypothesis that pain perception can be described according to the rules of Bayesian probabilistic reasoning. Emotions, memories, expectations and beliefs, together with sensory inputs, reflect the interplay of the system with the environment, its present state and its past experience. On the basis of these considerations and of our findings, we claim that these non-sensory components, far from being events that interfere with the pain perceptive process, are all evidences that the system uses—along with nociceptor spiking activity—to decide what is useful to perceive. In this way the system can end up with a more effective perception than using nociceptor signals alone, as suggested for other perceptive modalities [24]

We also argue that to fully understand the placebo effect we should consider it in the broader context of the general strategies that the system follows to bring forth pain perception. In the placebo effect, perception is biased by the cue. But what we call bias is the result of the integration of additional information, and the placebo effect would result from stimuli that are unlikely given the context. This differs from the conclusions of Morton et al. [71]. They too believe that pain perception may be considered probabilistic and described in a Bayesian framework, however they suggest that the placebo effect results from an ambiguity caused by prior expectation for analgesia. Instead, we consider the ambiguity intrinsic to sensory signals, and we show that expectation elicited by the cue may increase the probability of what, in its absence, would be less probable.

Finally, we think that the theoretical framework we explored may also provide an answer to the question about why the placebo effect exists. Not because it is useful by itself: what is useful is the logic underlying it. The placebo effect would be a perceptive illusion and, like other illusions, results not from a malfunction of the system but is a byproduct of computational strategies pursuing optimal integration of information [26, 33, 76]. The model we developed is an attempt to describe such a logic.
